# Impaired Economy of Gait and Decreased Six-Minute Walk Distance in Parkinson's Disease

**DOI:** 10.1155/2012/241754

**Published:** 2011-09-12

**Authors:** Leslie I. Katzel, Frederick M. Ivey, John D. Sorkin, Richard F. Macko, Barbara Smith, Lisa M. Shulman

**Affiliations:** ^1^Baltimore Veterans Affairs Medical Center and Geriatrics Research Education and Clinical Center, Baltimore, MD 20201, USA; ^2^Division of Gerontology & Geriatric Medicine, Department of Medicine, University of Maryland School of Medicine, Baltimore, MD 20201, USA; ^3^Department of Neurology, University of Maryland School of Medicine, Baltimore, MD 20201, USA; ^4^Maryland Exercise and Robotics Center of Excellence, VA Rehabilitation Research & Development, Baltimore, MD 20201, USA; ^5^University of Maryland School of Nursing, Baltimore, MD 20201, USA

## Abstract

Changes in the biomechanics of gait may alter the energy requirements of walking in Parkinson's Disease (PD). This study investigated economy of gait during submaximal treadmill walking in 79 subjects with mild to moderate PD and the relationship between gait economy and 6-minute walk distance (6 MW). Oxygen consumption (VO_2_) at the self-selected treadmill walking speed averaged 64% of peak oxygen consumption (VO_2_ peak). Submaximal VO_2_ levels exceeded 70% of VO_2_ peak in 30% of the subjects. Overall the mean submaximal VO_2_ was 51% higher than VO_2_ levels expected for the speed and grade consistent with severe impairment in economy of gait. There was an inverse relationship between economy of gait and 6MW (*r* = −0.31, *P* < 0.01) and with the self-selected walking speed (*r* = −0.35, *P* < 0.01). Thus, the impairment in economy of gait and decreased physiologic reserve result in routine walking being performed at a high percentage of VO_2_ peak.

## 1. Introduction

Walking capacity is central to the performance of many activities of daily living. Difficulty with walking is one of the cardinal symptoms of Parkinson's Disease (PD). Alterations in the biomechanics of gait, such as decreased stride length, increased stride length variability, and reduced gait speed, are common even in early stages of PD [1–3]. Most often, PD patients attempt to compensate for short steps by increasing gait cadence, thereby potentially altering energy requirements. This higher energy cost of movement is often referred to as a lower economy of gait and is a function of abnormal gait patterns that accompany aging and neurological disability. Reduced economy of gait has been associated with impaired function and fatigue in non-PD populations [4–9], but there is currently scant information on how parkinsonian gait affects energy expenditure or economy of gait using direct measures of oxygen consumption [10]. Further, little is known about the relationship between economy of gait and mobility. Hence, the purpose of this study was to investigate economy of gait during submaximal treadmill walking in mild to moderate PD, and the relationship between economy of gait and the distance covered during the 6-minute walk (6 MW).

## 2. Methods

### 2.1. Subjects

Participants for this study were recruited from the University of Maryland Parkinson's Disease Center and the Baltimore VA Medical Center neurology clinics as part of an exercise intervention trial in PD [11]. Inclusion criteria were (1) diagnosis of levodopa-responsive PD characterized by 2 of 3 cardinal signs (resting tremor, bradykinesia, rigidity), (2) Hoehn and Yahr (HY) [12] stage 1 to 3 (while “on” for motor fluctuations), and (3) presence of mild to moderate gait impairment, (score of 1 or 2 on Unified Parkinson's Disease Rating Scale (UPDRS) [13] questions no. 29 Gait or no. 30 Postural Stability, (4) Age ≥ 40, )5) Folstein mini-mental state examination [14] score ≥ 23, and (6) unlikely to require PD medication adjustment for 4 months. Exclusion criteria were (1) unstable cardiac, pulmonary, liver, or renal disease, (2) unstable hypertension or diabetes, (3) anemia, orthopedic, or chronic pain-restricting exercise, (4) unstable psychiatric illness, or (5) >20 minutes of aerobic exercise more than 3 times per week (to avoid prior training effect). This study was approved by the Institutional Review Board at the University of Maryland, Baltimore, and written informed consent was obtained from each participant. 

All physical performance measures, rating scales, and functional tests were performed while the subjects were “on” or within 3 hours of medication intake. Subjects used an additional dose of medication to maintain the “on” state when necessary.

### 2.2. Assessments

The UPDRS was administered by a neurologist with expertise in movement disorders (LS). The Total UPDRS includes three subscales: Mentation, Behavior, and Mood (Part I), Activities of Daily Living (Part II), and the Motor Examination (Part III). Short distance ambulatory function was assessed with three-timed 10 meter walks. The self-selected walking speed was defined as the average velocity of the three tests. This short-distance test is widely recognized as a valid index of mobility recovery and simulates the distance required for many home-based daily functions. The 6 MW is a distance that is more representative of community-based daily activities. Participants were instructed to cover as much distance as possible in 6 minutes, turning every 100 feet, as prompted by orange traffic cones set apart across a flat, clear space.

### 2.3. Exercise Treadmill Testing

**Screening Treadmill Test**

A screening graded-treadmill test to voluntary exhaustion without measurement of the rate of oxygen consumption (VO_2_) was performed using a manual protocol as previously described [15, 16]. All treadmill testing was performed in the early afternoon while the subjects were “on”. This screening exercise treadmill test served to (1) acclimate the subjects to walking on a treadmill (2) evaluate for symptoms of overt coronary disease or to detect silent myocardial ischemia (3) evaluate hemodynamic heart rate and blood pressure response to exercise (4) observe gait patterns and (5) determine whether there were any issues that would preclude their ability to safely exercise. All subjects wore a gait belt for safety, and a spotter stood behind subjects during the treadmill evaluations. Subjects were instructed to use the minimum level of handrail support for balance during the test.

The initial target speed for treadmill testing was the subject's self-selected over ground walking velocity, with the incline set at 0%. The first stage was conducted for 2 minutes at 0% grade, the next stage was conducted for 2 minutes at 4% grade, and then the grade was subsequently advanced by 2% every minute until voluntary exhaustion. In frailer subjects, the second stage was conducted at 2% instead of 4% for a more gradual increase in workload. Once the grade reached 10%, subjects were asked if the speed of the treadmill could be simultaneously advanced with grade (generally by 0.2 mph). The electrocardiogram (ECG) was monitored continuously, and blood pressure was measured during the first 3 stages of the tests and every 2 minutes during recovery.

**Exercise Treadmill Test with Measurement of Peak Oxygen Consumption**

At the next study visit one week later, subjects underwent a progressive-graded exercise treadmill test to voluntary exhaustion as described above with measurement of peak oxygen consumption (VO_2_ peak using a Quark Cardio Pulmonary Exercise Testing metabolic analyzer (Cosmed, Rome, Italy)). In some subjects, the initial treadmill speed was adjusted slightly based on the results of the screening treadmill test and feedback from the research subjects. As a result, the average self-selected walking speed on the treadmill was 94% of their self-selected over ground speed (2.31 ± 0.59 miles per hour (mph) versus 2.46 ± 0.53 mph). The first stage was conducted for 2 minutes at 0% grade (first submaximal treadmill stage), and then advanced as described above. O_2_ consumption, CO_2_ production, and minute ventilation were measured breath-by-breath, and values averaged for 20 second intervals. Subjects were instructed not to talk during the test as this is known to affect the depth of breathing and gas exchange. Based on our pilot study [15], we anticipated that we would not be able to measure true maximal aerobic capacity (defined as a plateau in oxygen consumption during the final stage, maximal heart rate >85% of age-adjusted predicted maximal heart rate, and respiratory quotient (RQ) or respiratory exchange ratio (RER) > 1.10) in many of these deconditioned subjects. The VO_2_ peak was based on the mean of the final two 20-second averages obtained during the final stage of the test.

### 2.4. Economy of Gait

We used the average O_2_ consumption values obtained over the final 40 seconds of the first sub-maximal treadmill stage to measure economy of gait. The 2-minute duration of this stage is similar to the time spent on many activities of daily living. Economy of gait was calculated as the measured VO_2_ during the first treadmill stage divided by the predicted VO_2_ for non-PD age-matched subjects based on commonly accepted American College of Sports Medicines equations for subjects walking accounting for treadmill speed and grade [17].

VO_2_ = horizontal component + vertical component + resting component,
VO_2_ (mL/kg/min) = 0.1 (speed) + 1.8 (speed) (fractional grade) + 3.5,
Speed = speed in meter/minute, to convert to mph, 1 mph = 26.8 meter/minute.

Higher oxygen consumption levels for any given speed and treadmill grade imply increased energy expenditure and impaired economy of gait.

### 2.5. Statistics

SAS version 9.2 (SAS Institute, Inc, Cary, NC, USA) was used for the statistical analyses. Descriptive statistics are expressed as mean ± standard deviation (SD). Pearson's correlation coefficients were used to calculate strength of relationship between variables. All statistical tests were two sided and performed at a significance level of 0.05.

## 3. Results

Seventy-nine subjects (57 men and 22 women) completed this cross-sectional study. Physical characteristics and PD severity scores are summarized in Table 1. Based on the UDPRS and HY ratings, the subjects had a broad range of disease severity from mild to moderately severe PD. Eleven subjects (7%) had received deep brain stimulation surgery for PD. The level of medical comorbidity in the sample was low, with only five individuals (6%) with prior history of stable coronary artery disease, seven (10%) on medication for diabetes, and only one was a current smoker (1%). Twenty-nine subjects (37%) were on medications for hypertension, including five on betablockers. 

**Figure 1:**
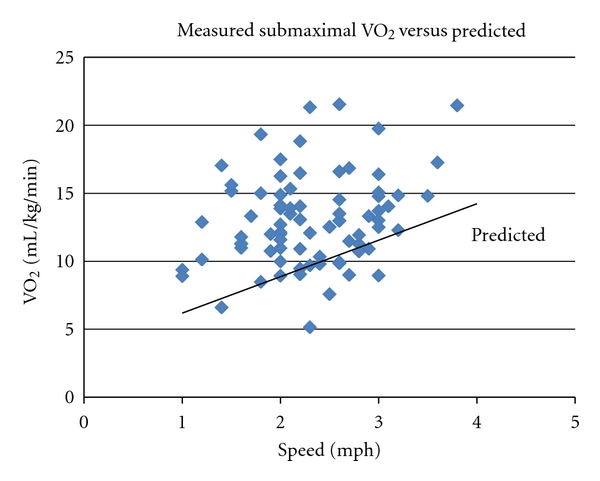
Submaximal VO_2_ measured at self-selected walking speed during the last 40 seconds of the first 2-minute stage of treadmill test versus walking speed in mph. Diamonds show measured values, where solid line shows expected value (VO_2_ mL/kg/min predicted = 0.1 × 26.8 speed in mph + 3.5). The vast majority of subjects had measured values higher than the predicted values indicative of poor economy of gait.

The VO_2_ at the self-selected treadmill walking speed averaged 64% of their VO_2_ peak. There were, however, a wide range of values (31% to 89% of VO_2_ peak). Interestingly, 24 of 79 subjects had submaximal VO_2_ levels that exceeded 70% of their VO_2_ peak, indicating severe reduction in economy of gait, with 3 subjects approaching 90% of their VO_2_ peak. Overall the subjects had mean submaximal, self-selected walking speed VO_2_ values that were 51% higher than the VO_2_ levels expected for the same speed and grade for non-PD subjects (13.0 ± 3.3 mL/kg/min versus 9.7 ± 1.6 m/kg/min). This observation provides clear evidence of the large decreases in economy caused by parkinsonian gait patterns (Figure 1). 

**Figure 2:**
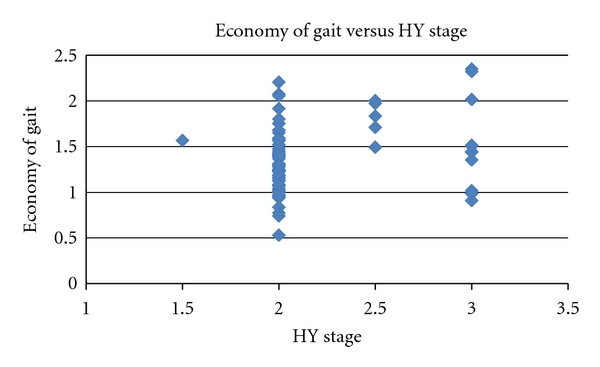
Relationship between the Hoehn and Yahr stage and economy of gait (ratio of measured VO_2_ to predicted VO_2_). Higher values of the ratio of measured VO_2_ to predicted VO_2_ are indicative of impaired economy of gait.

**Figure 3:**
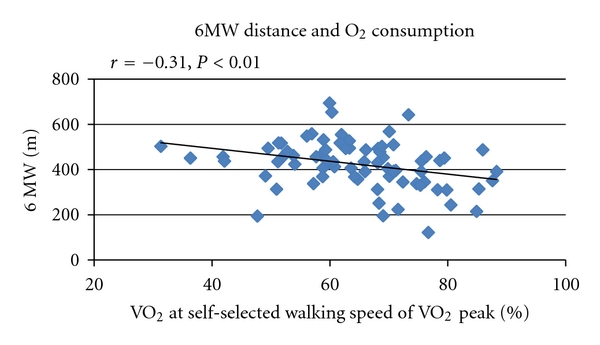
There was an inverse relationship between the distance covered during the 6 min walk (6 MW) and the amount of oxygen subjects consumed at their self-selected walking speed during the first stage of the treadmill expressed as a percentage of their VO_2_ peak.

We examined whether PD severity was associated with economy of gait (the ratio of measured VO_2_ and predicted VO_2_). There was a significant correlation of HY stage with economy of gait (Figure 2) with more advanced PD severity associated with lower economy of gait. There was no relationship between economy of gait with total or motor UPDRS. There was an inverse relationship between economy of gait and the distance covered during the 6 MW (*r* = −0.31, *P* < 0.01). Specifically, individuals whose measured VO_2_ was a higher percentage of their VO_2_ peak during their self-selected walking speed covered less distance walking for six minutes (Figure 3). There was also an inverse relationship between walking speed on the treadmill test and economy of gait (*r* = −0.35, *P* < 0.01).

## 4. Discussion

Our results demonstrate that economy of gait is markedly impaired in people with mild to moderate PD that increases the energy demands of physical activity. Our subjects walking at their self-selected pace on the treadmill required on average 64% of their VO_2_ peak. Indeed, 30% of our subjects used over 70% of their VO_2_ peak during their self-selected treadmill speed, and several subjects approached 90% of their VO_2_ peak. By contrast in healthy younger and older individuals, most activities require a small percentage of the maximal or peak working capacity as indexed by their VO_2_ peak [18, 19]. In a study of seniors without PD, the percentage of oxygen uptake (VO_2_/VO_2_ peak) during low, moderate and high workload levels was 32%, 42%, and 50%, respectively [20]. In that study, VO_2_ at the fastest comfortable walking speed was 40% of VO_2_ peak, values substantially lower than those observed in our subjects with mild to moderate PD. 

There was a relationship between HY stage and economy of gait, such that individuals with more severe PD had poorer economy of gait. Impairments in gait and mobility impact on the ability of subjects with PD to perform a number of gait-dependent daily activities including housework, dressing, and transferring in and out of bed [21]. Impaired gait economy may result from many factors including abnormal gait biomechanics and altered spatiotemporal aspects of gait associated with PD, that is, slow, short-stepped shuffling gait with decreased stride length, asymmetric arm [22] swing, tremor and rigidity, postural instability, loss of range of motion of axial structures, impaired sensorimotor integration, and so forth, The modest association between economy of gait and disease severity and economy of gait and distance covered during the 6MW test also indicates that other factors such as balance problems, difficulty with turning, and physical deconditioning contribute to impaired mobility in these subjects [1–3]. 

Few studies have directly measured walking economy in PD. Christiansen et al. examined walking economy at a number of walking speeds in subjects with PD compared to healthy subjects without PD [10]. VO_2_ was found to be 6 to 10% higher in people with PD at walking speeds above 1 mph. We report much greater impairments in walking economy than Christiansen et al. The VO_2_ for our subjects at 2 mph is 12.5 mL/kg/min, whereas Christiansen et al. reported a VO_2_ of 11 mL/kg/min at this speed. This difference may be explained by greater PD severity in our population (mean total UPDRS score, 47 versus 32). We used published equations for VO_2_ rather than direct measurement in a control population. The predicted VO_2_ for a given walking speed derived from younger individuals may underestimate the energy cost of walking in healthy older adults [19]. Protas et al. [23] also studied submaximal oxygen consumption during steady-state exercise in PD. Exercise performance was assessed using cycle ergometry in PD and non-PD. The PD group was unable to perform the same level of exercise as rated by maximum power when compared with the control group, even though the peak VO_2_ and heart rate were similar. The authors concluded that there was poorer exercise efficiency in the PD group than in controls. Over a range of submaximal cycling intensities, rates of energy expenditure were about 20% higher in PD than in controls. Thus, our results support previous findings of reduced economy of gait and exercise efficiency in PD.

There is growing interest in the effects of aging and medical comorbidities on bioenergetics and their impact on mobility and other measures of physical performance [18, 24]. We have previously reported that subjects with mild to moderate PD have VO_2_ peak values 20 to 25% lower than healthy age-matched controls [16]. This impairment in VO_2_ peak, in combination with the higher energy demands of walking (lower economy of gait), reduces the physiologic reserve in PD. The decreased physiologic reserve and lower VO_2_ peak make it more difficult to perform everyday tasks. The higher energy cost of walking necessitates the use of anaerobic pathways to meet ordinary energy demands, which may be associated with fatigue [18, 20]. Clearly, the decreased physiological reserve shown in this study has functional consequences as evidenced by impaired 6 MW distance and slow self-selected walking speed, particularly in those with more severe PD. The predicted 6 MW distance for healthy subjects without PD using the equation of Enrichi and Sherrill [25] that takes into consideration age, gender, height, and weight was 509 meters compared to the measured 424 meters, a difference of 85 meters, or 17% lower in PD. The 6 MW distance in our subjects is comparable to the values reported by Falvo and Earhart [26] who reported a 6 MW distance of 394.1 ± 98.4 m in PD patients of similar age to our subjects.

This study has limitations that may result in an underestimation of the severity of the impairment of economy of gait. (1) The submaximal O_2_ utilization was measured by using O_2_ utilization during the last 40 seconds of the first stage of the treadmill test, when subjects walked at their self-selected speed and 0% grade. We chose this time as representative of the time period in which our subjects typically walked. A number of investigators have advocated measuring submaximal O_2_ for longer periods of time [7, 18, 27]. For example, Alexander et al. measured O_2_ kinetics in frail and non-frail older adults during a 6-minute submaximal exercise bout on the treadmill [7]. The Baltimore Longitudinal Study of Aging employs a 5-minute stage, but the data from the first 1.5 minutes is discarded [18]. This allows for a longer period of time for the subjects to come to equilibrium and plateau during the bout of submaximal exercise. We recognize that it is possible that some of subjects did not plateau during the second minute of the exercise due to a lag in O_2_ uptake at the start of exercise reflecting impaired O_2_ kinetics. However, any error introduced would have biased our measuring less O_2_ utilization as subjects with delayed O_2_ kinetics would take longer to come to equilibrium [7, 27]. Hence, we potentially understated the degree of inefficiency of our patient sample with respect to economy of gait. (2) Another limitation is that the O_2_ consumption during exercise includes a resting component for the resting metabolic rate. Indeed this resting component is included in the American College of Sports Medicine equation [17]. This resting component is often measured with the subjects in the supine position [28], but others have advocated measuring it by having the subject stand for 5 minutes prior to the walking test [9] as this allows an examination of the incremental O_2_ utilization attributable to the exercise itself. The resting metabolic rate in subjects with PD might be affected by age-related changes in body composition, sarcopenia, as well as other changes attributable to PD (i.e., resting tremor and medication effects). Changes in resting metabolic rate in PD may be clinically significant as a higher resting metabolic rate is associated with increased mortality in older adults [28]. Even if the increased metabolic needs during exercise are partially explained by an increased resting metabolic rate, the net effect on ambulatory function is the same; more energy is needed for a given level of ambulation. (3) Another potential confound is the use of handrail support during this study. Subjects were instructed to walk on the treadmill with minimal hand support. Subjects varied in the extent to which they used the side rails for balance support. The use of hand support reduces O_2_ consumption, again leading to a possible underestimate of their O_2_ utilization (VO_2_) and subsequent underestimate of the degree of impairment of their economy of gait. (4) These measures were performed with subjects walking on treadmills. Frenkel-Toledo et al. have proposed that treadmill walking may act as an external pacemaker to improve gait variability [29]. If gait biomechanics improve on the treadmill, this would reduce oxygen utilization and lead to an overestimate of their economy of gait. The gait biomechanics and energetics might be different in overground walking. (5) Lastly the 6-minute walk test required subjects to make tight turns around a cone. This might have adversely impacted the distance covered, particularly in subjects that had limited ability to turn, that is, turning “en bloc”. Future studies employing portable metabolic systems could be employed to examine economy of gait during overground walking.

There is substantial interest in whether the abnormalities in gait and functional performance in PD can be improved by treadmill exercise training [30–32]. In a pilot study by Pelosin et al. [32], 10 patients with idiopathic PD underwent 4 weeks of treadmill training (30 min, three times a week for 4 weeks). Walking performance (Timed Up and Go, 6-min and 10-m walking tests) and metabolic function (oxygen uptake and heart and respiratory rate) were evaluated before training, at the end of treatment and after 30 days with two different graded exercises (treadmill and cycle ergometer). Training significantly improved walking performance. Oxygen uptake, and heart and respiratory rates were significantly decreased only during graded exercise on the treadmill but not on the cycle ergometer consistent with improved economy of gait, but the data are difficult to interpret due to the way they are displayed in the paper.

In summary, this study reinforces prior evidence showing impaired economy of gait in PD that is associated with impairment of ambulation at both short and long distance. Reduced economy of gait combined with the reduced VO_2_ peak results in lower physiologic reserve where even comfortable gait is performed at a high percentage of VO_2_ peak. Future research should examine the biomechanical and neuromuscular factors that contribute to impaired walking economy in PD. A better understanding of these factors may lead to new approaches to improve functional performance and quality of life in PD.

## Figures and Tables

**Table 1:** Subject characteristics, disease severity, and physical performance measures.

Parameter (*N* = 79*)	Mean ± SD	Range
Age (years)	65.1 ± 10.7	42 to 86
UPDRS total	47.2 ± 14	15 to 96
UPDRS motor	32.4 ± 10.2	11 to 66
Hoehn and Yahr stage	2.2 ± 0.4	1.5 to 3.0
Hoehn and Yahr Stage 1.5	*N* = 1 (1%)	—
Hoehn and Yahr Stage 2.0	*N* = 61 (77%)	—
Hoehn and Yahr Stage 2.5	*N* = 5 (6%)	—
Hoehn and Yahr Stage 3.0	*N* = 12 (15%)	—
Body mass index (kg/m^2^)	28.1 ± 4.9	18.0 to 41.6
VO_2_ peak (mL/kg/min)	22.4 ± 4.8	12.6 to 37.4
Submaximal VO_2_ (mL/kg/min)	13.0 + 3.3	5.1 to 21.6
Walking speed (mph)	2.31 ± 0.59	1.0 to 3.8
6 min walk distance (meters)	424 ± 106	122 to 695

*6-min walk performed in 75 subjects.
